# Ambulance nurses’ experiences as the sole caregiver with critical patients during long ambulance transports: an interview study

**DOI:** 10.1186/s13049-024-01178-1

**Published:** 2024-01-23

**Authors:** Jenny Wästerhed, Erika Ekenberg, Magnus Andersson Hagiwara

**Affiliations:** Centre for Prehospital Research Faculty of Caring Science, Work Life and Social Welfare Boras, University of Borås, Borås, Sweden

**Keywords:** Ambulance services, Critical patient care, Rural areas, Prehospital emergency care, Nursing

## Abstract

**Background:**

Working in rural areas involves tackling long distances and occasional lack of supportive resources. Ambulance nurses are faced with the responsibility of making immediate autonomous decisions and providing extended care to critically ill patients during prolonged ambulance transport to reach emergency medical facilities. This study aims to expose the experiences of ambulance nurses acting as primary caregivers for critically ill patients during lengthy ambulance transfers in rural regions.

**Method:**

Fifteen nurses employed in an ambulance service within sparsely populated rural areas were subjected to semi-structured interviews. The collected data underwent qualitative content analysis.

**Result:**

The analysis resulted in one overarching theme with two categories. The theme is ‘Safety in the Professional Role,’ and the two categories are ‘Working in sparsely populated areas presents challenges’ and ‘Rare events: when routine cannot be established.’ The findings suggest that working as an ambulance nurse in a rural setting poses various challenges that can be highly stressful. Delivering care to critically ill patients during extended ambulance transports requires the knowledge, experience, and careful planning of the healthcare provider in charge.

**Conclusions:**

The findings underscore the necessity for thorough planning and adaptable thinking when attending to critically ill patients during extended transport scenarios. The absence of supporting resources can render the task demanding. Nevertheless, participants reported an inherent tranquility that aids them in maintaining focus amid their responsibilities.

## Background

Long ambulance response times, coupled with extended care durations during transport and the delayed seeking of care by rural patients for their symptoms, contribute to poorer prognosis for many patients in sparsely populated areas compared to their urban counterparts [1, 2]. Notably, there is a significant disparity in arrival times and ambulance care duration between rural and urban areas [3]. For nurses working in ambulance services in sparsely populated regions, the substantial distances between pickup locations and emergency hospitals, as well as road conditions, result in prolonged care periods during ambulance transport [4].

Various definitions exist for rural and semi-urban rural areas. The Swedish Agency for Growth Policy Evaluations and Analyses defines rural areas as those where it takes more than a 45-minute drive to reach a locality with at least 3000 inhabitants, while semi-urban rural areas are defined as those within a 45-minute drive of urban areas with 3000 inhabitants or more [5]. The Nomenclature of Territorial Units for Statistics (NUTS) classification, used to classify socio-economic areas in the EU and the UK, categorizes areas as rural if they have fewer than 12.5 inhabitants per square kilometer and as very sparsely populated if the population falls below 8 inhabitants per square kilometer [6]. In Sweden, this includes most inner parts of Norrland, archipelago islands, and parts of Småland. Alanazy et al.‘s study [3] revealed an average response time difference of seven minutes between urban and rural ambulances, with urban areas having an average arrival time of about 15 min, compared to rural areas’ 22 min.

Ambulance missions with critically ill patients during extended ambulance transports to the nearest emergency hospital demands a high level of competence. Prehospital care must be tailored to the patient’s current medical condition and the urgency of their needs [7]. Emergency medical service (EMS) clinicians need extensive knowledge across various caring areas and practical familiarity with technical equipment to ensure effective and safe care delivery.

Most EMS clinicians express confidence in their ability to work under pressure and rapidly assess prehospital patients [8]. The care environment can quickly become stressful, chaotic, and demanding, requiring nurses to remain alert and prepared for unexpected developments [7]. Additionally, EMS clinicians must stay updated on current guidelines [9].

In cases of trauma, the involvement of physican-staffed resources enhances the prehospital survival rates of patients [10]. The absence of such resources can leave EMS clinicians without assistance for extended periods, leading to concerns about losing control of the situation and prolonged waits for support from assisting units [4, 11]. In addition to the delay in receiving assistance, stress can affect decision-making, with EMS clinicians often forced to make quick decisions under time constraints and uncertain circumstances, increasing the risk of making incorrect choices [4]. This study aims to expose the experiences of ambulance nurses acting as primary caregivers for critically ill patients during lengthy ambulance transfers in rural regions.

## Method

### Design

The study utilized a qualitative inductive design involving interviews with Swedish ambulance nurses.

### Selection and participants

The study was conducted in a central Swedish county that encompasses both urban and rural areas. This county has a population of 285,000 residents, including one large city, one medium-sized city, two smaller cities, and several urban areas, spanning a total area of 18,000 square kilometers. The county’s ambulance service is comprised of ten ambulance stations. For this study, four ambulance stations were included, one located in a smaller urban area and three in rural areas. All four of these stations serve predominantly sparsely populated regions.

Permission for the study was sought from the operations manager in the respective area. Once approval was granted, ambulance unit managers at the selected ambulance stations were contacted to provide information and obtain the necessary permits. Initially, two individuals working at an ambulance station in a county with sparsely populated areas were approached to participate in the study. They were then asked to recommend additional participants, resulting in a total of 15 nurses from the four ambulance stations located in sparsely populated or small urban areas being interviewed. There was significant interest in participating, and participants were selected through a combination of subjective selection and a snowball sampling approach.

The inclusion criteria for participants were registered nurses, with or without specialist education, who work in ambulance service in sparsely populated areas. To ensure a wide range of perspectives, there were no restrictions on the number of years of professional experience for study participants. Emergency Medical Technicians (EMT) were excluded. EMTs have a shorter (one-year) training in prehospital emergency care and are not intended to care for critically ill patients anddid not align with the study’s objectives. In Sweden, since 2005, registered nurses have been responsible for patient care and medication management [12].

The study included six male and nine female participants. On average, participants had 8.4 years of experience in rural ambulance work (see Table 1). Nine participants had received one or more specialist education, while eight had not.

**Table 1:** Demographics of study participants

Gender Age	Year as Licensed Nurse	Specialist educated	Years within ambulance	Median (Range)	Years within rural area	Median (Range)
Men 45,0	13,5	67%	14,1	9 (0,4–27)	12,6	5 (0,4–26)
Women 40,75	10,3	56%	6,1		5,6	
Average 42,4	11,6	60%	9,3		8,4	

### Data samples

The interviews were semi-structured and consisted of open-ended questions. An interview guide comprising six main questions served as the initial framework. These questions included inquiries such as: “*What is your experience like working in an ambulance stationed in sparsely populated areas?”* and “*Could you share an instance when you provided care to a critically ill patient during an extended transport?*” To test the interview guide and the interview technique, a preliminary interview was conducted, and this interview was also incorporated into the study.

Given the prevailing Covid-19 pandemic, participants were offered the option to conduct the interviews via an internet connection with both audio and video, which was chosen by four participants. Eleven participants opted to be interviewed at a location of their own choosing, ensuring compliance with current guidelines considering the ongoing pandemic.

### Analysis

The collected material was processed using Graneheim and Lundman’s content analysis method [13] to identify patterns and subsequently categorize them. The recorded interviews were repeatedly listened to and transcribed verbatim by the respective authors. Transcription of the interviews was carried out as soon as possible after the interviews to preserve any conveyed impressions, including emotional expressions or body movements. Two authors then read through the transcribed interviews multiple times to gain an overall understanding of the collected material’s content [14]. It’s worth noting that the first authors have a substantial prior understanding of the topic at hand, given their extensive experience as specialist nurses in ambulance healthcare in sparsely populated areas. The pre-understanding can be both negative and positive. Pre-understanding may lead to an overinterpretation of the transcribed interviews but can also contribute to increased reflexivity in both the interview situation and in the analysis. The study’s findings were continuously discussed with the study’s third author, who has less experience in rural ambulance healthcare.

To ensure objectivity and prevent the imposition of their values and impressions, the authors engaged in critical discussions of each other’s interview material with a questioning approach. More follow-up questions were utilized to elucidate participants’ experiences further, minimizing the risk of interpretational errors stemming from the authors’ preconceived notions. The analysis encompassed both manifest and latent content analysis [13]. After comprehensively reviewing the material, meaningful units were identified, condensed to capture the essence, and irrelevant text was omitted These condensed units were then coded and categorized into various subcategories. Similarities and differences were identified, and textual quotations were employed to illustrate the content. The first two authors conducted the analysis together, constantly discussing the meaning of the text and how best to describe it in categories., resulting in the emergence of two categories, six subcategories, and an overarching theme.

To validate the findings, three nurses from different healthcare professional backgrounds were invited to review and provide feedback on the work. This step was taken to receive constructive comments and insights regarding whether individuals from various professional categories comprehended the chosen area, even without prior knowledge of the prehospital care environment.

## Result

The results are presented in two categories comprising a total of six subcategories. An overarching theme emerged.

### THEME:

Safety in the Professional Role.

- **Working in sparsely populated areas presents challenges.**
  - The shortage of resources entails more than just a lack of personnel.
  - Technological issues can complicate decision-making.
  - Long transports of critically ill patients necessitate careful planning.
- **Rare events: when routine cannot be established.**
  - Collaborative teamwork among colleagues is of paramount importance.
  - The capacity to maintain inner composure in stressful situations.
  - Education enhances job security.

### Summary of the theme safety in the professional role

The results indicate that working as an ambulance nurse in a rural ambulance comes with numerous challenges that can be quite stressful. Providing care to critically ill patients during extended ambulance transports necessitated the knowledge, experience, and careful planning of the healthcare provider in charge. The quality of care was constantly put to the test, given the frequent limitations in terms of support and access to additional ground ambulances or helicopter services.

Despite these obstacles, the participants were able to summon an inner sense of calm by concentrating on their immediate tasks. They found that by making the most of the available resources, they could cultivate a sense of confidence in their work. However, this level of confidence was not something achieved overnight. Inexperienced ambulance nurses often leaned on their more seasoned colleagues for support, ultimately contributing to their sense of professional security.

### Working in sparsely populated areas presents challenges

In sparsely populated areas, the road conditions were not consistently good, and unexpected obstacles arising from weather conditions and strong winds often prolonged both the travel and transportation time to the hospital. Even when specific missions were dispatched, the crew was aware from the outset that time was of the essence, and adverse conditions could further complicate the situation for the patient.

Possessing thorough local knowledge was seen as a distinct advantage for urgent assignments because the response time was frequently lengthy, and time pressure remained a constant factor. Proficiency in local geography facilitated decision-making by allowing for the selection of optimal routes that could significantly reduce the time required to reach the patient, ultimately enhancing the ambulance nurses’ confidence and patient care. Participants with limited knowledge of the local terrain experienced stress when they couldn’t rely solely on GPS map references.

Less experienced participants struggled with uncertainty regarding which hospital to transport the patient to, given that not all hospitals were equipped to handle emergencies. This made it challenging to determine the appropriate destination based on their current location.

*“Yes, time is working against us, you can feel it… mostly you talk about the golden hour and the golden… here and there, but you can already sense it slipping away when you step out…”*.

There could be misunderstandings in the communication between the ambulance and the Emergency department (ED) regarding handover. ED staff sometimes encountered patients who, while not critical at the moment they arrived, had been in critical condition at the pick-up location. These patients had received treatment during the extended transport, and by the time they reached the hospital, their vital signs had stabilized. However, there remained a high risk that these patients could deteriorate once again. Some of the participants found it challenging to convey this information during the handover process because the ED staff typically assessed patients based on their current status. Many participants felt that they had to justify their reports when delivering patients to the ED, which was both frustrating and difficult. Working in a sparsely populated rural area in an ambulance not only presented challenges in caring for critically ill patients over extended periods but also posed difficulties with patients whose conditions were not initially classified as life threatening. These patients might have appeared stable at the beginning of the journey, but during the extended transport to the hospital, their condition could rapidly deteriorate.

*“So it might seem like the work is quite distinct; we have time for both… we can even have enough time to treat a critically ill patient to the point where they are no longer in critical condition when we hand them over at the hospital. Then, you find yourself in the position of having to explain to the receiving unit that the patient was actually critical from the outset, which they don’t always comprehend because they perceive the patient as they are at this moment.”*

### The shortage of resources entails more than just a lack of personnel

All participants found the experience of working in sparsely populated ambulances to be both enjoyable and stimulating, despite the challenges they encountered. Those who had worked in both urban and sparsely populated areas noted significant differences. In urban areas, there was a greater availability of resources that could arrive quickly to provide assistance. However, in sparsely populated areas, this same level of assistance was not readily available. Participants with more extensive experience often expressed a need for additional hands to help with patient care in the ambulance. Conversely, those with less experience described feeling isolated and solely responsible for making critical decisions in patient care. The frustration of having limited resources was perceived as making the work environment more challenging. When colleagues arrived on the scene, particularly in situations where neither a helicopter nor another ambulance was available, those with less experience felt a profound sense of relief.

The shortage of resources extended beyond the availability of additional ambulances, helicopters, or colleagues at the accident scene; it also pertained to the lack of time for reflection. Participants believed that they should have been allocated time for reflection on various incidents, but this often had to be postponed due to the constraints of their work hours. Participants with limited ambulance experience felt that there was insufficient time for reflection. They expressed a desire for scheduled debriefing and reflection sessions after the end of their shifts, as these sessions would have provided valuable learning opportunities and allowed them to enhance their knowledge and skills.

*“Usually, it’s very intense with alarms… and just now… you’ve responded to an alarm, perhaps you want to return to the station to contemplate and discuss… this is what we used to do, could we have handled it better… could we have done this or that… to improve ourselves as newcomers… and especially, maybe the veterans also want to embrace something new… but that time is unavailable (with emphasis)… there is no time for it. I feel… I might view it somewhat negatively… because all reflection, whether you only transported a patient or not, is valuable… for both sides…”*.

### Technological issues can complicate decision-making

A complicating factor encountered when working in rural ambulances was technological issues. For instance, there were instances where the global positioning system (GPS) provided incorrect route suggestions due to impassable roads. Mobile networks were either unavailable or incapable of transmitting information to another healthcare unit for assistance, such as interpreting electrocardiograms (ECGs).

The absence of mobile coverage for consulting with medical support made the sense of isolation palpable. The long distance to the hospital and the expectation of an extended treatment period necessitated making decisions entirely independently. Handling a critically ill patient in such circumstances posed a significant challenge, and the participants emphasized the importance of having confidence in their professional role.

When they were able to reach out to medical support, it was seen as a valuable resource for obtaining advice, guidance, and confirmation that their thinking aligned with that of the physician. However, when caring for critically ill patients, the participants found that the usual medical support they turned to did not always meet their specific needs, particularly in cases of trauma. Contacting the appropriate medical specialists, facilitated decision-making.

Time constraints were frequently experienced when dealing with critically ill patients in these situations. The awareness of the long distances an ambulance could travel during a mission in sparsely populated areas affected the participants. They felt responsible for not unnecessarily tying up resources, even though they acknowledged this was an organizational issue. Those with more experience sometimes hesitated longer than their less experienced counterparts to request a helicopter or transport patients over long distances. Keeping an ambulance occupied for several hours was undesirable, so they might delay calling for a helicopter or additional road ambulance unless the patient’s condition was undeniably critical. The participants felt a duty to ensure that resources remained available for patients in genuine need.

*“At times, I’ve found myself in the middle of the forest, with no signal on the radio, making it impossible to reach medical support or the on-call doctor at the hospital… This means I have to make more decisions on my own, so it’s crucial to feel confident in my professional role… Especially when we have a lengthy period of patient care… and then… when they are critically ill patients, it can be challenging. It takes longer to obtain assistance, and it’s more challenging to coordinate a rendezvous on the way to the hospital… you end up working more independently in some ways.”*

### Long transports of critically ill patients necessitate careful planning

The participants in the study frequently had extended transport times with patients. They believed that this necessitated careful planning of how to administer treatment. When caring for critically ill patients over an extended duration in an ambulance, the actions taken had time to produce effects before reaching the hospital, and the actions not taken could potentially impact the patient. Having a multi-stage plan was a crucial aspect of their work.

Planning for tasks during demanding patient transports presented a challenge. Medical supplies could be depleted without the possibility of replenishment, requiring creative problem-solving and the use of available materials as part of the job.

Experience and training were invaluable and supportive factors during long transports with critically ill patients. They instilled confidence in decision-making and care planning during transport. Individuals with less experience heavily relied on the equipment and the knowledge and experience of their colleagues. Effective communication between team members was of paramount importance.

Given the potential for unexpected situations to arise, having an alternative plan ready was essential. Being proactive and considering various risks during extended transports was deemed more manageable with adequate education, a broad knowledge base, and accumulated experience. These three factors facilitated the work and contributed to ensuring patient safety. Although extended transport times were often viewed as a challenge, they were also seen as positive opportunities for treatment and interventions to take effect during transit, potentially leading to the patient’s improvement before arriving at the receiving emergency department. It was evident that these interventions made a positive difference for the patient, resulting in a gratifying sense of accomplishment.

*“Given the nature of the job, one must constantly strive to improve their actions using rudimentary tools and find a balance in their usage… because of the long distances we cover, you have to anticipate several steps ahead, considering what challenges you might encounter during the journey… It’s probably simpler to apply pressure to a major bleeding wound with a cloth when you’re near the hospital, but if you’re far away from the hospital, you might need to think beyond that cloth and consider what other actions can be taken?”*

### Rare events: when routine cannot be established

A “rare event” was described as a significant task occurring at infrequent intervals, demanding a high level of competence from the caregiver because the necessary knowledge had to be readily available. Caring for critically ill children was deemed a rare event, as was managing an obstructed airway.

Nurses required an extended period to accumulate experience in these challenging assignments due to their rarity. Simultaneously, it was the experience gained from caring for critically ill patients during lengthy transports that played the most pivotal role in enabling work to be carried out with minimal stress. For newcomers to rural ambulance work, the absence of experience in such assignments made their colleagues the most valuable resource in reducing stress.

During rare events, ambulance nurses felt a heightened sense of security when other ambulance nurses were present because they could easily discuss the patient’s condition and medication, given their shared profession. Particularly, those new to the ambulance service experienced relief when other units arrived on the scene, allowing them to share or transfer responsibility to a more experienced ambulance nurse. The arrival of a helicopter at the accident scene was seen as a positive development since the helicopter crew included a doctor who assumed medical responsibility for the patient.

Rare events were experienced as challenging and stressful, but it also offered an opportunity to gain experience and knowledge for future assignments. It heightened self-awareness regarding how to function in stressful situations, thus reducing the stress associated with encountering a similar event in the future. Post-assignment discussions were considered highly important, not only to identify areas for improvement but also to validate the work that had been performed.

*“It was a comforting sensation… I wasn’t immobilized, and I was able to function. I could take action and provide treatment to the child following CPR guidelines and other trauma protocols. Knowing that I can operate in such a situation for the next time. Having the self-assurance that I can perform effectively.”*

### Collaborative teamwork among colleagues is of paramount importance

Working in an ambulance involved long-term partnerships, sometimes in challenging situations without assistance from other units. To manage the demanding and revealing work with critically ill patients in sparsely populated areas, feeling supported by one’s colleague was crucial for establishing a sense of security. Having a colleague with whom participants could discuss and brainstorm ideas was considered highly valuable.

Effective communication played a vital role in collaborative work. When both colleagues achieved situational awareness and worked towards the same objectives, it significantly facilitated their tasks. It was evident that colleagues played a substantial and important role, especially for those with less education and experience, as they provided significant support. The experience largely depended on the colleague with whom the ambulance nurse partnered. Colleagues who exuded confidence enhanced the sense of teamwork, emphasizing the importance of seamless collaboration among colleagues.

In the ambulance, there were various professional categories with different levels of education. Some colleagues might not fully understand each other’s professional roles, and questioning a colleague’s decisions could be detrimental. When collaboration did not function optimally among colleagues, feelings of vulnerability and isolation became palpable. This sense of occasional solitude in making decisions and taking a stance existed regardless of the ambulance nurses’ professional backgrounds.

*“I was questioned by colleagues afterward… why I opted not to request a helicopter… but it was so far away that… and then you have to consider various factors… closely examine the timing and final destination. If that helicopter transport doesn’t take off, we would have lost valuable time… and considering the weather conditions at the time… it wasn’t feasible to request a helicopter, but… Yes, it was quite challenging.”*

### The capacity to maintain inner composure in stressful situations

In sparsely populated areas with long distances, much of the work was often solitary, and a consistent experience was that the work was frequently isolating yet liberating. Participants encountered situations with critically ill patients where they had to provide care alone, and this was seen as stressful. However, they also felt an inner sense of calm that enabled them to concentrate on their duties and provide care for the patient.

Despite being aware of limited material and assistance resources, there was a determination to make the best of the situation at hand. Those with less experience often experienced greater stress during critical events and did not feel as calm as those with several years of experience.

In cases where an ambulance was not able to receive assistance from another healthcare unit while caring for a critical patient, the feeling of isolation could be pronounced, even if other units like emergency services were called to the scene. Maintaining inner calm amidst a stressful environment and situation allowed them to stay focused on the task and deliver high-quality care to the patient.

*“It was stressful, yet simultaneously there was that inner calm… I’m doing everything I can right now, there’s nothing more I can do… but, at the same time, it’s incredibly stressful, and you just wish you could let go of everything and walk away. It’s like you’re gazing at the edge of the forest, and it would be so much nicer to just walk away from here. You question why you’re subjecting yourself to this.”*

### Education enhances job security

Roughly half of the participants held one or more specialized certifications. Those with more specialized training possessed expertise in ambulance care and another specialty, and they felt that their distinct areas of specialization complemented each other. Their education and professional experience in fields beyond emergency medical care gave them a sense of security in various situations.

Participants with anesthesia and ambulance specialist training described having knowledge that instilled confidence when dealing with challenging airway management and the care of unstable patients.

For ambulance nurses, regardless of their length of experience, lacking experience or training in pediatric care was stressful. Participants with both ambulance and pediatric specialist training felt secure when transporting critically ill children because of their prior experience. Those with both general primary care and ambulance training believed it benefited them since most transports involved elderly patients. They also felt that they had a foundational understanding of pediatric care, which boosted their confidence in such assignments.

In general, all ambulance nurses with one or more specialized certifications reported increased confidence and reduced stress, regardless of the specific combination of specialist training they had. Those with less experience felt more at ease working with colleagues who had one or more specialist certifications than with other inexperienced colleagues.

Training was viewed positively by all participants, and the less experienced individuals without specialist training expressed a desire for more training. However, their primary focus was on accumulating experience through daily work first. After gaining more experience, they planned to pursue additional education. New nurses in ambulance care were uncertain about which specialized training to choose. Several participants expressed interest in obtaining Intensive Care and/or Anesthesia training in addition to their Ambulance specialist training, as they believed this combination would provide a deeper understanding of working with critical patients.

All nurses who had completed prehospital care courses such as Prehospital Trauma Life Support or Advanced Medical Life Support felt that it provided a strong foundation for their daily work. Those who lacked or had only occasionally received concept training expressed a strong desire to receive this training. Without previous experience and concept training, it was challenging to arrive at a traffic accident scene and take on the role of medical officer. Newcomers also found it difficult to assert themselves and decline roles they didn’t feel prepared for. Feeling confident in their colleagues’ knowledge base reassured the inexperienced nurses that they were not alone.

Regardless of their experience and education level, everyone at some point encountered situations requiring difficult decisions, which could be overwhelming and mentally taxing. The most challenging aspects couldn’t be practiced despite all the training, leaving them largely at the mercy of the situation. Rare events, such as unexpected situations with a challenging work environment and numerous critically injured individuals, were cited as particularly demanding.

*“You have to be prepared for how conditions can evolve and have an understanding of that. It’s about the fact that these are rare events. Sparsely populated areas are often linked to a low volume of assignments, so you have to plan for yourself, I believe…”*.

*“Had I known how challenging this could be…then I’m not so sure I would have readily agreed to it… but perhaps that’s fate… maybe that’s why I’m here… because I was blissfully unaware of how tough it can be… how intricate the work is…”*.

## Discussion

In sparsely populated areas, the distance between the location where the ambulance picks up the patient and the emergency hospital where the patient is transported is often considerable. This means that EMS clinicians must care for critically ill patients for extended periods within the ambulance. Long distances can exacerbate the patient’s condition [15] and increase the risk of mortality compared to urban areas [16]. Practicing prehospital care in an ambulance requires mental preparedness for unforeseen situations [17]. It is not guaranteed that assisting units will be available or within a reasonable distance when an ambulance responds to a critical patient [18]. As a result, EMS clinicians sometimes find themselves caring for the patient alone during transportation to the emergency hospital, even though having another colleague would be more convenient. They feel that everything relies on them, and they bear a significant responsibility to ensure safe and high-quality care for the critical patient. This heightened level of responsibility contributes to increased stress levels among ambulance staff [18].

Healthcare profession, in general, is widely regarded as one of the most stressful [19]. Ambulance work poses a risk of developing post-traumatic stress syndrome (PTSD) among staff [20, 21]. During call to serious events, EMS clinicians experience a surge of stress characterized by an increased heart rate and elevated cortisol levels in their blood [22]. This discomfort can persist long after a serious event and negatively affect work [23]. Having prior experience in demanding situations provides with a sense of safety during patient transport [24]. However, previous experiences in challenging situations can also trigger stress when facing similar events again [18]. The participants felt that there was a lack of dedicated time for reflection, with debriefing sessions occurring almost exclusively after major events. Inexperienced participants expressed a desire for similar sessions after less serious events. Having the opportunity to debrief was highly valued by ambulance clinicians [25].

The term “rare events” emerged during the interviews to describe infrequent situations that are difficult to gain experience in but require a high level of competence due to their sporadic occurrence. Particularly stressful were situations involving critically ill children. Even non-critically ill children, when cared for during lengthy transports, could induce stress. If the children were seriously ill and EMS clinicians had to care for them without assistance available, stress levels could escalate. Caring for critically ill children is considered one of the most demanding and stressful situations that paramedics encounter in their work [18, 25, 26].

The participants in this study emphasized the importance of understanding limited resources and the necessity of concentrating on the current task, making the most out of each situation. Various strategies are employed to deal with stressful events [27]. Contextual awareness in a situation leads to adaptive strategies, such as prioritizing the task over emotions [28]. The significance of exercises and training in high-pressure situations was underscored by the participants. This can enhance the sense of manageability, facilitating EMS clinicians functioning in challenging work scenarios [27]. Access to a physician’s support was generally seen as beneficial, except in critical patient cases when participants felt there was insufficient time to contact a physician, and the physicians support often lacked familiarity with the ambulance nurse’s work environment. In a study on interhospital patient transports, the ability to reach someone for problem-solving discussions was deemed highly valuable, particularly when the supporting physician was familiar with the ambulance environment and its conditions [24].

Other stress-reduction factors included effective collaboration with colleagues [18]. Newly hired ambulance nurses experienced a strong sense of security when working alongside experienced colleagues. Having a trustworthy, experienced colleague was deemed essential for newly employed ambulance nurses [25]. The absence of assistance from other ambulance units underscored the critical importance of collaboration with a colleague. When team communication did not function optimally, the work became more challenging, potentially jeopardizing patient safety. Communication breakdowns within a team are a significant contributing factor to medical errors [29]. Achieving situational awareness collectively and working toward a common patient-focused goal became a considerable challenge. Effective teamwork and communication are vital, particularly in challenging situations, to uphold patient safety [30].

### Limitations

By interviewing ambulance nurses with varying levels of education and experience, the authors had the opportunity to illustrate the ambulance nurses’ experiences of caring for critically ill patients during extended transports from different angles. One potential factor that may have negatively affected the study is that it was conducted during a pandemic with mask mandates in place, which could have impacted the interpretation of results as facial expressions could only be partially perceived. Additionally, some of the interviews were conducted via computer connection rather than in-person meetings for the same pandemic-related reasons, potentially leading to the loss of nuances conveyed through body language and gestures during the interviews. Furthermore, the study only included one region in Sweden. Nevertheless, the study still achieves transferability as it encompasses several ambulance stations.

## Conclusions

The study reveals that rural ambulance services pose significant challenges for staff, leading to isolation and heightened stress during extended care for critically ill patients. Professional confidence is bolstered by prior experience and education. Trust in colleagues becomes crucial when resources are scarce or when nurses lack training. Reflective time is vital for addressing challenges, especially for less experienced participants.

Key findings emphasize the importance of comprehensive education and knowledge for EMS clinicians in sparsely populated areas. Training for handling “rare events” is crucial for patient safety. The study provides diverse perspectives by interviewing ambulance nurses with varying levels of education and experience.

Given limited research on rural ambulance services, further exploration is needed. Future studies could focus on training, knowledge enhancement, assessments for lone ambulance nurses, and stress management strategies during extended transports. Recommendations include dedicated reflection time for new employees, mandatory hospitalization days for organizational understanding, and internal prehospital care training for a safer environment and enhanced patient safety.

## Data Availability

The datasets used and/or analysed during the current study are available from the corresponding author on reasonable request.

## Abbreviations

NUTS: The Nomenclature of Territorial Units for Statistics
EMS: Emergency medical service
EMT: Emergency Medical Technicians
ED: Emergency department
GPS: Global positioning system
ECG: Electrocardiogram
PTSD: Post-traumatic stress syndrome
